# Acute respiratory distress syndrome following administration of gadolinium contrast agent: a case report

**DOI:** 10.1186/s13256-022-03643-w

**Published:** 2022-11-17

**Authors:** Eduardo Atsushi Osawa, Juliana Fernandez Fernandes Kleiman, Alexandre Toledo Maciel

**Affiliations:** 1 Imed Group Research Department, Av. Angélica, 2.530 - 9º andar, São Paulo, SP 01228-200 Brazil; 2 Adult Intensive Care Unit, São Camilo Hospital, Pompeia Unit. Av. Pompéia, 1178, São Paulo, SP 05022-001 Brazil

**Keywords:** Gadolinium, Acute respiratory distress syndrome, Anaphylaxis

## Abstract

**Background:**

Gadolinium-based contrast agents are used extensively in magnetic resonance imaging to assist diagnosis of medical conditions. Despite their documented safety profile, severe adverse events do occur, and their documentation may serve to raise the awareness of the medical community.

**Case presentation:**

We report the case of a 15-year-old white Latin American female patient admitted to the intensive care unit for acute respiratory distress syndrome following administration of gadolinium. She did not have rash or tongue swelling but developed hypotension responsive to fluid administration and severe hypoxemia. Chest computed tomography revealed bilateral pulmonary compromise with multiple confluent consolidations. She received methylprednisolone and noninvasive ventilatory support including bilevel positive airway pressure ventilation and high-flow nasal cannula, and underwent a rapid recovery.

**Conclusion:**

Gadolinium-based contrast agent-induced acute respiratory distress syndrome, albeit rare, should be included in the differential diagnosis of respiratory failure shortly after magnetic resonance imaging, which is nowadays a frequent diagnostic procedure, potentially increasing the awareness of this serious complication.

## Introduction

Gadolinium-based contrast agents (GBCAs) are employed widely in magnetic resonance imaging (MRI) to assist the diagnosis and follow-up of numerous medical conditions. It is estimated that 50 million doses are injected annually, and side effects are rarely reported [1]. Despite extensive documentation on the safety profile of GBCAs, severe adverse events occur and may serve to raise the awareness of the medical community. A rare but serious adverse event related to GBCA administration is the development of acute respiratory distress syndrome (ARDS), which is a life-threatening, extensive, and diffuse inflammatory process of the lungs with damage of the alveolocapillary barrier leading to noncardiogenic pulmonary edema, gas exchange impairment, and severe hypoxemia [1]. In this case, ARDS was the terminal complication of GBCA administration rather than the primary event. Thus, we present this case of a patient who developed ARDS after GBCA administration.

## Case presentation

A 15-year-old white Latin American female patient underwent contrast magnetic resonance imaging (MRI) for outpatient investigation of persistent pelvic pain. In a recent emergency department (ED) visit for the same complaint, a normal urinalysis was obtained. Her past medical history was unremarkable, and she did not take any medications and denied alcohol, tobacco, or any drug abuse. Few minutes after receiving gadolinium during MRI, she presented to the ED with shortness of breath, chest pain, dizziness, nausea, and vomiting. Upon admission, she had a brief period of loss of consciousness and her vital signs were as follows: blood pressure (BP), 84/40 mmHg; temperature, 36.4 °C; heart rate (HR), 122 beats per minute; respiratory rate (RR), 30 breaths per minute, and peripheral oxygen saturation (SpO_2_) of 78%. Her pulmonary auscultation revealed bilateral rales, but she did not have rash, or tongue or lip swelling. Despite significant hypoxemia, she was able to speak in whole sentences, and inhaled oxygen was delivered through a non-rebreather mask (NRBM) at 10 L per minute. She immediately received 500 mg of hydrocortisone and a 1 L bolus of intravenous crystalloids, but epinephrine was not administered upon the patient’s presentation. Also, antimicrobial therapy with ceftriaxone and azithromycin was initiated.

Laboratory tests demonstrated a hemoglobin level of 18.6 g/dL, a slightly elevated white blood cell count (15,360/mm^3^, normal 4000–11,000/mm^3^), an increased C-reactive protein (CRP) concentration (41.8 mg/L, normal: < 0.5 mg/L), a lactate dehydrogenase (LDH) level of 176 U/L (normal 135–214 U/L), and a d-dimer concentration of 1.7 mg/mL (normal: < 0.5 mg/mL). B-type natriuretic peptide and troponin levels were low, and the echocardiogram was normal.

**Fig. 1:**
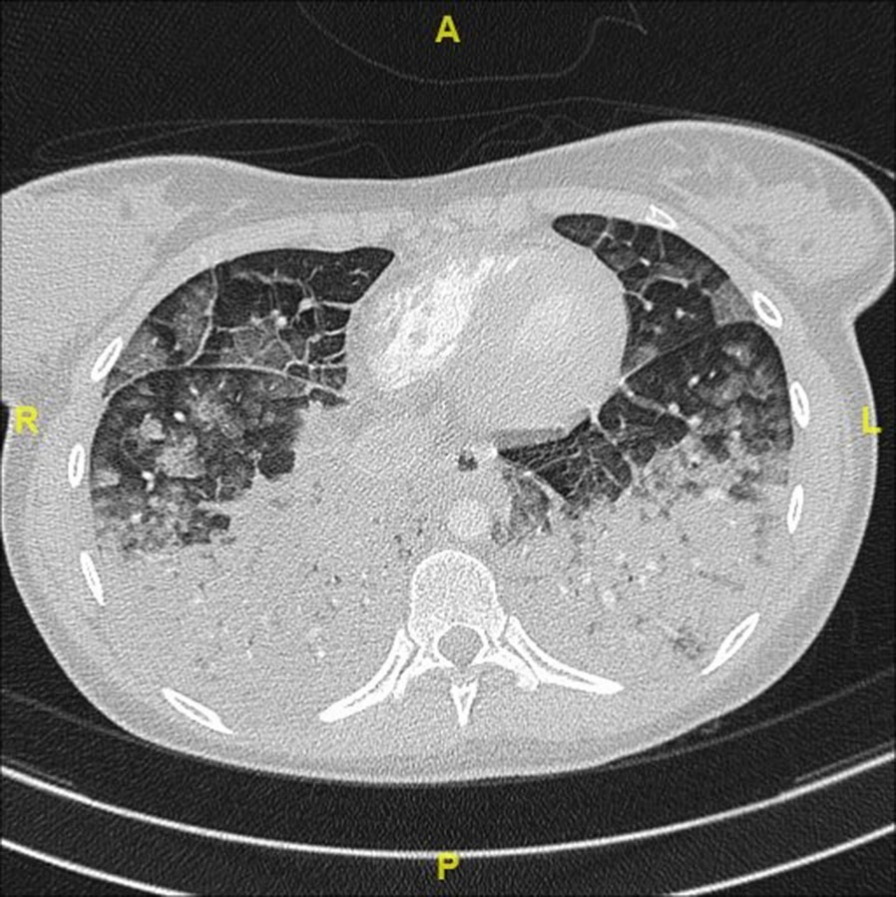
Chest computed tomography (CT) showing bilateral multiple confluent consolidations located mainly in the posterior aspects of the lower lobes associated with regions of ground-glass opacities in the upper lobes and diffuse thickening of interalveolar septa

A chest computed tomography (CT) was performed around 1 hour after GBCA administration and showed bilateral pulmonary compromise secondary to multiple confluent consolidations located mainly in the posterior aspects of the lower lobes. There were also regions of ground-glass opacities that were more evident in the upper lobes along with diffuse thickening of interalveolar septa (Fig. 1). A CT pulmonary angiography ruled out the diagnosis of pulmonary embolism. A severe acute respiratory syndrome coronavirus 2 (SARS-CoV-2) nasopharyngeal swab testing came back negative.

She was then transferred to the intensive care unit (ICU) with improved vital signs: BP, 119/71 mmHg, HR, 122 beats per minute, and SpO_2_ of 93%. Due to high oxygen requirement, methylprednisolone 1 mg/kg was prescribed and she was commenced on bilevel positive airway pressure (BiPAP) ventilation and high-flow nasal cannula (HFNC). The patient showed substantial improvement over the following 12 hours, which enabled her to be weaned off noninvasive ventilatory support. She was afebrile during the entire hospital stay. She was discharged from ICU on day 2 receiving inhaled oxygen via nasal cannula at 1 L per minute and discharged home on day 3, with SpO_2_ of 98% on room air. Owing to the patient’s rapid recovery after supportive treatment and the absence of clinical evidence of infection, the decision to cease antibiotic therapy was made. Corticosteroid treatment was tapered down slowly over 30 days after discharge. Six months after initial presentation, the patient remained well and the follow-up was uneventful.

## Discussion

We report the case of a young female patient who developed a life-threatening complication after GBCA administration. This case is unique as respiratory distress and hypotension occurred immediately after infusion, there was no skin involvement, and the symptoms improved rapidly with noninvasive supportive therapies.

The advance of imaging technology in recent decades has led to a substantial increase in GBCA use. It is estimated that 50 million doses are injected annually, and side effects are rarely reported [2]. As compared with iodine-based contrast agents, GBCAs are associated with a lower incidence of acute reactions [3] and guidelines on their safe use have primarily focused on the risks for renal toxicity and development of nephrogenic systemic fibrosis [4]. GBCA-associated adverse events can be classified into two categories: nonallergic reactions (headache, dizziness, nausea, vomiting, pain at the injection site) and idiosyncratic allergy-like reactions [5]. Despite growing awareness of GBCA-related complications owing to increased MRI use, severe life-threatening reactions are rare and were reported to range from 0.1% to 0.001% [6, 7].

Drug-induced acute lung injury is a challenging diagnosis in clinical practice. There is no specific biomarker for ARDS triggered by GBCA, and the clinical presentation is indistinguishable from other causes. Therefore, the etiology is suspected by the temporal relationship between drug injection and development of symptoms, along with the exclusion of other risk factors. The pathogenesis is unknown; however, explanatory mechanisms have not been linked to systemic anaphylaxis but to chemical endothelial injury triggered by activation of the complement system [8]. This results in the exudative phase of ARDS characterized by accumulation of protein-rich fluid within the interstitium and alveolus [1, 9].

We identified 11 cases of ARDS associated with GBCA administration reported in literature. In contrast to our patient, fever was reported by some investigators [10–12] and was deemed to be a feature of the ARDS presentation. Patients differed with regard to disease severity, with supportive respiratory support varying from noninvasive therapies [10–14] to invasive mechanical ventilation [15–18] and venoarterial extracorporeal membrane oxygenation (VA-ECMO) [19]. In two patients [17, 19], cardiac arrest was documented, but they recovered after cardiopulmonary resuscitation (CPR) and supportive therapies. Notably, in our patient, we used a noninvasive respiratory support strategy identical to that described by Nguyen [12] comprising noninvasive ventilation (NIV) and high-flow nasal cannula (HFNC), with a similar outcome. Interestingly, aside from the similarities that this patient presented with our case, including age group, gender, and reason for MRI, they also had hemoconcentration upon ED arrival. This finding may be associated with fluid extravasation into the interstitium and alveoli, and in both cases, hemoglobin levels normalized after clinical improvement.

In only two patients [10, 17], the onset of symptoms occurred immediately after contrast administration, a finding similar to our case report. In other patients, the duration from GBCA injection and symptom onset ranged from 10 minutes to 3 hours. In a study comprising six prospective observational multicenter studies, 82.4% of patients developed acute drug reactions within 5 minutes after GBCA administration, and the majority of the study population did so within 15 minutes [7]. In fact, despite the absence of skin involvement, our patient fulfilled the diagnostic criteria for anaphylaxis [20] given the presentation of hypotension and respiratory distress following contrast administration. Measurement of trypase levels could have assisted in the differential diagnosis, however we were unable to obtain this during hospitalization. As in other cases, our patient received supportive therapies and a course of corticosteroid treatment. She showed rapid clinical improvement without requiring either vasopressor therapy or advanced respiratory support modalities. Because sepsis is the major cause of ARDS, antibiotics were administered initially, but unfortunately no blood cultures were obtained. Urinalysis was normal in a recent ED admission, fever and shivering were absent, and a modest CRP increment was observed. Moreover, the absence of signs of sepsis or respiratory failure before gadolinium administration and the temporal relationship between GBCA infusion and symptom onset suggest that ARDS was not triggered by infection.

## Conclusion

GBCA-induced ARDS is a rare but potentially serious complication and should be included in the differential diagnosis for respiratory failure shortly after MRI. Since gadolinium use is very frequent in the current medical diagnostic armamentarium, this adverse event is expected to be more frequently observed. No specific treatment has been advocated, and supportive therapy seems to be the cornerstone for these cases. Methylprednisolone may eventually be useful, but its real utility remains to be defined.

## Data Availability

The data used to support the findings of this case study are included within the article.
